# Quality-of-Life Assessment in Pediatric Advanced Cancer: Development of the Patient-Reported Outcome Measure *Advance QoL*

**DOI:** 10.3390/curroncol31040170

**Published:** 2024-04-19

**Authors:** Lye-Ann Robichaud, Julie Felipe, Michel Duval, Bruno Michon, Marianne Olivier-D’Avignon, Sébastien Perreault, Mathias Tyo-Gomez, Marc-Antoine Marquis, Serge Sultan

**Affiliations:** 1 Department of Psychology, Université de Montréal, Montréal, QC H3C 3J7, Canada; lye-ann.robichaud@umontreal.ca (L.-A.R.); julie.felipe@umontreal.ca (J.F.); 2 Azrieli Research Centre, CHU Sainte-Justine, Montréal, QC H3T 1C5, Canada; s.perreault@umontreal.ca; 3 Department of Pediatrics, Université de Montréal, Montréal, QC H3C 3J7, Canada; michel.duval@umontreal.ca (M.D.); marc-antoine.marquis@umontreal.ca (M.-A.M.); 4 Department of Hematology-Oncology, CHU Sainte-Justine, Montréal, QC H3T 1C5, Canada; 5 Centre Mère-Enfant Soleil, CHU de Québec-Université Laval, Québec, QC G1V 4G2, Canada; bruno.michon@chudequebec.ca; 6 School of Social Work, Université Laval, Québec, QC G1V 0A6, Canada; marianne.davignon@tsc.ulaval.ca; 7 Department of Neurology, CHU Sainte-Justine, Montréal, QC H3T 1C5, Canada; 8 Psycho-Oncology Center (CPO), CHU Sainte-Justine, Montreal, QC H3T 1C5, Canada; mathiasgomeztyo@gmail.com; 9 Department of General Pediatrics, CHU Sainte-Justine, Montréal, QC H3T 1C5, Canada

**Keywords:** pediatric cancer, advanced cancer, palliative care, quality of life, measure

## Abstract

A recent measure was developed to assess the Quality of Life (QoL) of young people with advanced cancer and is available for parents and professionals (*Advance QoL*). The present study aimed to elaborate self-reported versions for children and adolescents with advanced cancer. We adopted a four-phase research plan: (1) to elaborate the *Advance QoL* questionnaire for youth (8–12 and 13–18 years old) with a team of young research partners; (2) to evaluate the understandability of these versions in a sample of 12 young patients from the target population using cognitive interviews; (3) to assess social validity in the same group using a questionnaire and the content validity index (CVI); and (4) to refine the questionnaires according to these results. Four major themes were identified: (1) issues affecting the understanding of the tool; (2) issues that did not affect the understanding of the tool; (3) modifications to improve the tool; and (4) positive features of the tool. *Advance QoL* was well received, and feedback was positive. Adjustments were made according to young people’s comments and two self-reported versions are now available. It is essential to measure the key domains of QoL in advanced cancer. *Advance QoL* self-report versions will help target the specific needs of young people with this condition and their families.

## 1. Introduction

In oncology, advanced cancer is predominantly used to describe conditions for which standard curative options are exhausted. In North America and high-income countries, about 17% of children and adolescents with cancer will live with an advanced cancer, but in many parts of the world this figure is much higher (e.g., 50% in Latin America) [1,2,3]. Young people with advanced cancer can benefit from pediatric palliative care (PPC), a philosophy and approach to medical care which aims to alleviate pain and other symptoms, assist with decision-making, alleviate suffering, and promote Quality of Life (QoL) for patients and their families [4,5,6]. In this context of care, QoL should be regularly and systematically assessed to identify patients’ needs [7,8].

QoL is generally understood as a multidimensional concept underpinned by a subjective first-person evaluation. Therefore, measuring QoL requires evidence-based patient-reported outcome measures (PROMs) to evaluate the perception of their own status. The regular use of PROMs in clinical settings can have positive impacts on communication between patients and healthcare professionals, and on patient satisfaction towards their care and health [9,10]. This allows QoL to be monitored and issues to be detected that otherwise would go unnoticed [9,10].

In advanced cancer specifically, strong arguments support the use of PROMs to evaluate QoL among young patients. PROMs provide invaluable information to guide intervention when the emphasis is on optimizing comfort [11]. Patients may take such tools as an opportunity to discuss their symptoms and their QoL and express their healthcare preferences [5,12,13,14]. It also may help them mitigate the experienced stress and the feeling of isolation [15,16,17,18].

Contrasting with this need, two recent systematic reviews have shown that existing QoL assessment tools have several limitations when used with young people with advanced cancer [19,20]. These limitations may regard the content of the tools, with the recall period being considered inappropriate for young people, or the lack of coverage of important QoL-related domains. Critiques have also been addressed of their development, including a lack of involvement of young people, parents, and healthcare providers, or limited psychometric properties [19,20].

To address these issues, researchers have been developing a practical measure to assess QoL in situations of serious cancer without a cure [21,22,23]. This measure, named *Advance QoL*, is based on a definition of QoL elaborated from qualitative studies with young people, their parents, and healthcare professionals [21,22]. Seven important QoL domains were identified and characterized by specific indicators: physical, psychological, social, pleasure, autonomy/independence, pursuit of achievements, and feeling heard. A study has reported the refinement of a proxy measure appropriate for adult respondents (e.g., family members, professionals) [22]. Yet, the language level of this version is too complex for children and adolescents requiring a college reading level (Scolarius French readability index) [24].

**Figure 1:**
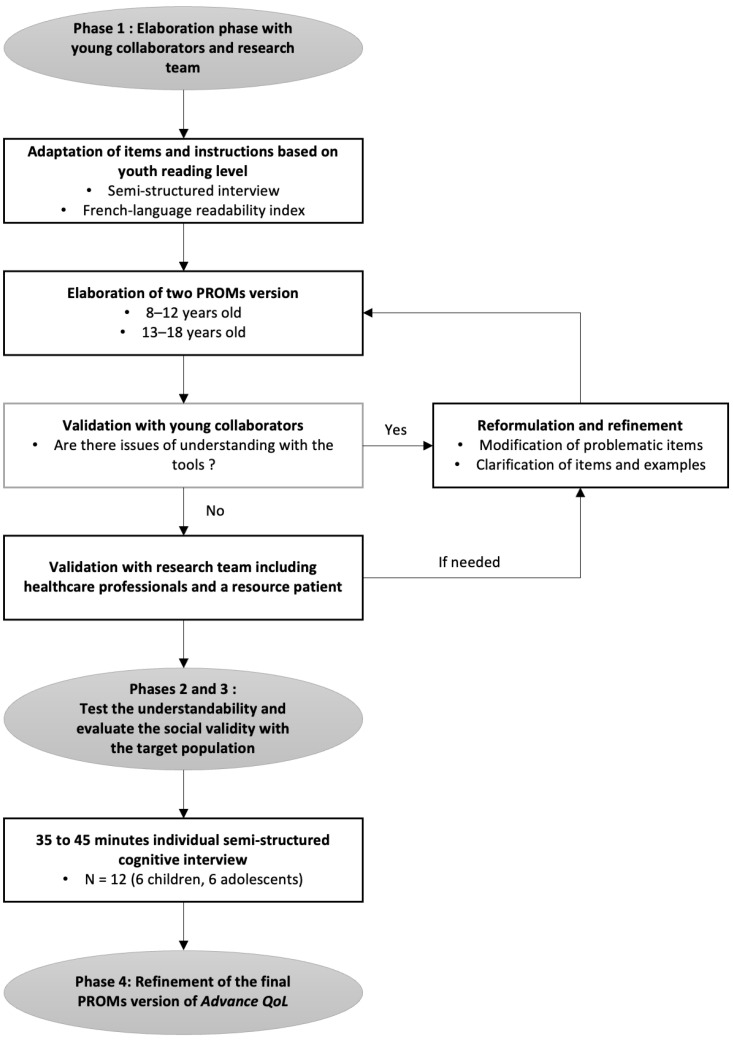
The development process of *Advance QoL* self-reported versions.

The present study aimed to adapt this proxy version of *Advance QoL* to be used by children and adolescents as a self-report. To guide this process, the project was divided into four distinct objectives to be explored in four phases (Figure 1):

1. Elaborate preliminary versions of the *Advance QoL* questionnaire for youth aged 8–12 years and 13–18 years;
2. Test the understandability and clarity of these versions in the target population;
3. Evaluate the social validity, i.e., the acceptability, pertinence, and satisfaction of these two versions;
4. Adapt and refine the versions of the questionnaire based on the previous results.

## 2. Materials and Methods

### 2.1. Phase 1: Elaboration

The elaboration of versions of *Advance QoL* for 8–12 year olds and 13–18 year olds involved an iterative process. Age ranges were established based on other QoL assessment tools [19,20,25,26]. The research team collaborated with five young healthy people (4 girls) aged 9–16 years (see Acknowledgments). Individual semi-structured interviews with these young collaborators allowed us to develop versions adapted both in language and format to the targeted age groups. For example, we asked them “How would you word the text so that it would be easily understood by someone your age?” When needed, we reformulated and refined unclear items and examples. Clarity was monitored using the Scolarius French readability index (Influence Communication, Montréal, QC, Canada) [24]. A score of <89 (reading level of an 8 year old) was targeted for the 8–12 years version, whereas a score between 90 and 112 (reading level of a 12-year-old) was targeted for the 13–18 years version. The refinement continued until reaching the targeted scores. The adequacy of the versions was confirmed by the young collaborators. The versions underwent further review by the broader research team, including healthcare professionals and a resource patient, leading to additional changes. Final working versions were approved by the young collaborators. Those versions were then tested in subsequent study phases.

### 2.2. Phases 2 and 3: Evaluation of the Understandability and the Social Validity 

#### 2.2.1. Participants and Recruitment

Children and adolescents with cancer were recruited at the CHU Sainte-Justine cancer care centre, and in collaboration with Leucan, a non-profit organization, between December 2022 and July 2023. Participants had no pre-existing relationship with the data collection team. Eligible patients were aged 8–18 years, had been diagnosed with cancer at least 3 months prior, were receiving cancer treatment, understood French and could communicate verbally, and had access to an electronic device with Internet. Initially, the eligibility criteria targeted young people with advanced cancer. However, recruitment challenges led to expanding the criteria. Considering the specific objectives of this study, which focus on understanding the tool, we believed broadening the inclusion criteria would not affect the data collected. We used a purposive sampling method with maximum variation to ensure clinical and sociodemographic diversity among participants [27]. We expected that heterogeneous sampling would give access to a range of viewpoints to identify as many issues as possible with *Advance QoL* [28,29]. No exclusion criteria were used. 

Eligible patients were identified by healthcare team members or our partner organization Leucan. Two methods were used: (1) Healthcare team members briefly introduced the study to patients and parents, and those interested signed a consent form, allowing contact by a research team member (LAR, graduate psychology student) who provided detailed study information. (2) The healthcare team provided a list of eligible patients who were contacted by the same person via telephone or in person at the hospital while the family was waiting for an appointment. Written parental consent and patient assent were obtained before data collection. Participants received a CAD 50 gift certificate. The interviews were conducted by the first author, recorded, and subsequently transcribed. The study received ethical approval from the hospital research ethics committee (#MP-21-2022-3550). 

#### 2.2.2. Data Collection

Data were collected through virtual semi-structured cognitive interviews lasting 30 to 45 min [30]. The interview focused on the version of the tool corresponding to the participants’ age group (8–12 years or 13–18 years). We developed an interview guide using closed- and open-ended questions to assess comprehension of instructions, items, and examples (Supplementary File S1). Verbal probing was employed when needed for deeper insight [30,31], and participants were encouraged to suggest improvements for the tool. We wanted participants to focus on their comprehension of the tool rather than their answers. Therefore, to minimize the burden, the participants did not actually complete *Advance QoL* [31]. Pilot interviews with two young healthy collaborators from Phase 1 were conducted to ensure the smooth running of subsequent interviews.

Participants also completed a short social validity questionnaire inspired by Kazdin [32] and Manne et al. [33]. The assessment of social validity informs about the acceptability level, the importance of, and the degree of satisfaction with a procedure [34,35]. The questionnaire included 10 questions with a five-point scale from 1 (totally disagree) to 5 (totally agree) about the overall clarity of *Advance QoL*, its pertinence and its utility, the ease of utilization, and the satisfaction toward the tool (sample item: “The tool is useful for assessing my well-being.”). As suggested by Yusoff [36], participants were encouraged to provide verbal feedback, especially if they answered ≤3 (disagree or neutral), to better understand their perspective and opinion, and to facilitate the refinement. Sociodemographic and clinical data were collected in a brief questionnaire including age, sex, gender, education level, years since diagnosis, and pediatric cancer condition. 

#### 2.2.3. Data Analysis

**Cognitive interview.** We adapted Knafl et al.’s [31] protocol for the analysis and interpretation of cognitive interviews for instrument development. This method allowed us to identify issues and to make systematic decision about keeping, deleting, or revising items of *Advance QoL*. Items or features that were understood and interpreted consistently among participants were kept, while others were revised or abandoned. 

First, transcripts were generated from audio recordings of each interview. The analysis focused on identifying issues and potential improvements for *Advance QoL*. Therefore, we used an item-by-item analysis which allowed us to produce a worksheet report collecting the extracts from each participant interview relating to each element of the questionnaire. This method facilitated an exploration of similarities and differences in participants’ comprehension. Second, from the report produced, we identified and classified facilitators and issues reported by participants as well as suggestions for improvement. Categories were clearly defined to avoid ambiguity and were based on the literature or generated inductively. To ensure the reliability and integrity of the analysis, two team members (LAR and JF) independently coded the interviews. 

**Social validity questionnaire**. To ascertain the content validity of the social validity questionnaire, we used the content validity index (CVI) [36]. A CVI ≥ 0.83 with 6 experts indicates that the social validity questionnaire’s items are representative and relevant to the targeted constructs, i.e., acceptability, pertinence, and satisfaction [36]. Participants—youth with cancer—are considered experts in our study to assess and critique the social validity of *Advance QoL* as a self-reported tool. We calculated the three indices suggested by Yusoff [36] to systematically analyze CVI: (1) item-level content validity index (I-CVI), that is, the proportion of experts judging items as relevant; (2) scale-level content validity index based on the average method (S-CVI/Ave), that is, averaging all I-CVI; (3) scale-level content validity index based on the universal agreement method (S-CVI/UA), that is, the average of items that received a universal agreement between experts. Median and range were used to analyze the social validity questionnaire. A score of ≤3 would indicate a need for refinement. 

### 2.3. Phase 4: The Final Adaptation

Based on the Phase 2 and 3 results, we refined the *Advance QoL* self-reported questionnaires. To ensure the accuracy of our analyses and subsequent decisions, we presented the refined questionnaire versions to the young collaborators from Phase 1 and to a specialized educator from our hospital (see Acknowledgments). Based on the feedback received, we adapted the final versions of *Advance QoL*. 

## 3. Results

### 3.1. Phase 1: Elaboration Process

Following the steps detailed in the methods, we developed two preliminary versions (for 8–12 and 13–18 year olds) based on the proxy version [22]. As for the proxy, both versions used a descriptive and individualized approach, a three-point response scale, and a 24 h timeframe. *Advance QoL* allowed patients to describe their QoL according to seven domains in quantitative and qualitative manners. The questionnaire ends with a radar chart summarizing patients’ perceptions of their QoL. Semi-structured interviews with young collaborators made it possible to obtain alternate formulations that were easily understood by young people. For example, medical terms were removed or adapted, and generic or abstract terms were avoided. We used concrete words as used in everyday life by young people. During the iterative process, the Scolarius French readability score of each version decreased significantly, indicating a decrease in the required reading level [24]. For the 8–12 years and the 13–18 years versions, the Scolarius reading level scores decreased from 67 to 56 and from 72 to 57, respectively. The main difference between both versions was the use of pictograms in the 8–12 years version.

### 3.2. Phases 2 and 3

#### 3.2.1. Sample Characteristics

From December 2022 to July 2023, six children (4 girls) aged 8 to 12 years (M = 9.67) and seven adolescents (2 girls) aged 13 to 18 years (M = 15.83) were recruited at CHU Sainte-Justine and in collaboration with the community organization Leucan (Table 1). One adolescent withdrew from the project after completing the consent form, due to hospitalization and loss of interest in the project. Five participants were followed by the CHU Sainte-Justine pediatric palliative care team, and seven had a central nervous system tumour. School levels ranged from second grade to pre-university programs.

#### 3.2.2. Cognitive Interviews

We conducted a semi-structured cognitive interview with each participant (average duration: 36 min). Our interview guide focused on participants’ understanding of the various components of the self-reported questionnaires. Verbal probes were also formulated to gain insight into the barriers and facilitators of questionnaire comprehension. In the broader perspective of the Cognitive Testing Process [30], our focus was primarily on participants’ comprehension, with less emphasis on other stages of the cognitive process, such as recall, decision-making, and response processes. Therefore, the “item-by-item” method [31] enabled us to systematically identify barriers and facilitators to understanding the *Advance QoL* questionnaires. Agreement between the two team members (LAR and JF) who coded the interviews was very good. As expected, there were no differences in the feedback received between young people with advanced cancer and those undergoing cancer treatment. Four major themes were identified among the points raised by participants (Table 2): (1) issues affecting understanding of the tool; (2) issues that do not affect understanding of the tool; (3) modifications to improve and enhance the tool; and (4) positive features of the tool. These four themes were divided into 14 codes, which were used to define the nature of the difficulties encountered or to clarify the positive elements of the questionnaires. To better illustrate the kind of feedback received from the participants, examples of verbal statements are given in Table 2 for each code. All codes were clearly defined and mutually exclusive. Some codes were defined based on the literature, such as “unclear reference”, which has been defined as “a lack of precision and clarity as to the elements on which to base one’s answer” [31]. Other codes were generated inductively, such as “organization of instructions”, which refers to elements of instructions that facilitate completion of the tool (e.g., words in bold type, the instructions’ structure, or the reminder). All themes were present in both age groups, but some codes were found in only one age group, such as critiques on the instruction being too long, the lack of precision, the instruments containing too many examples, and the recall period. One additional theme was drawn from elements observed by the interviewer during interviews with the younger subjects: items requiring thought and a response beyond the cognitive capacities of the respondents. These results guided the adaptation and refinement of the *Advance QoL* self-reported questionnaires. 

Participant feedback varied by age group. Adolescents’ concerns focused more on the meaning of the questions and the intentions behind the items, while children were more concrete, and reported misunderstanding words. In this regard, the preliminary version of *Advance QoL* provided the label and definition of each QoL domain. These labels were derived from scientific jargon provided by caregivers (e.g., “physical”, “psychological”, “achievement”). For children, the meaning of those terms was unclear.

#### 3.2.3. Social Validity Questionnaire

Table 3 shows descriptive data for each item of the social validity questionnaire. The results of the CVI indices indicated that the members of both age groups agreed to judge the *Advance QoL* self-reported questionnaire as clear, relevant, useful, and acceptable (full results available in Supplementary Table S1).

### 3.3. Phase 4: The Final Adaptation

Overall, *Advance QoL* was well received, and feedback was positive. Only minor adjustments were required according to young people’s comments. The final versions are available in the study repository [37]. In the 8–12 years version, concepts were adapted using child-friendly language, and the answer box and instructions were simplified for easier completion. For the 13–18 years version, we mostly clarified wording to enhance the understanding of the questionnaire. Table 4 shows examples of changes made based on the results from Phases 2 and 3. These final versions were revised and accepted by the young collaborators from Phase 1 and a specialized educator from our hospital.

## 4. Discussion

This study aimed to elaborate two PROM versions (8–12 years, 13–18 years) of *Advance QoL* designed to assess the QoL of young people with advanced cancer. The research process consisted of four phases. First, we developed preliminary versions for the two age groups based on a proxy version with young collaborators, keeping the original spirit of the tool [22]. Second, we tested understandability in the target population using cognitive interviews to provide insights on issues that might otherwise have gone unnoticed [30]. The involvement of children and adolescents in the project provided high-quality feedback and a unique perspective. The results suggest that cognitive interviewing is a rich method for children and adolescents with cancer to collect understandability data. In line with the objectives of this study, we gave importance to all comments raised by the participants. Each point raised in connection with a barrier, or a positive feature of the questionnaire, was categorized. We identified four major themes based on the participants’ feedback: (1) issues affecting understanding of the tool; (2) issues that do not affect understanding of the tool; (3) modifications to improve and enhance the tool; and (4) positive features of the tool. 

Although each theme was raised by children and adolescents, differences between the age groups underscored the necessity for two versions of the tool, considering their distinct stage of cognitive maturity, language, and abstract thinking. 

The children’s concerns highlighted the necessity to use simple and concrete words commonly used and known by young people. This result is consistent with what is suggested in the literature [38]. However, it can be challenging to identify difficult words and the appropriate reading levels for young people. Therefore, in addition to cognitive interviews, we used readability scores which indicate the required reading level to access a text [24]. Among the issues reported by adolescents, we found that some issues were due to our wish to oversimplify wording. The adolescents’ reflections, sometimes very elaborate, challenged the meaning of certain items. For some adolescents, the oversimplification of language was detrimental to their understanding, because essential elements to understanding the meaning of the item were lost (e.g., see “Unclear reference” in Table 2). Thus, readability scores were relevant for assessing text comprehension but proved insufficient for evaluating the understandability of formulations in the refinement process.

Furthermore, significant differences were observed between children and adolescents regarding parental roles and involvement. During adolescent interviews, none needed support from a parent, while most of the children did. In these cases, we informed parents that the purpose of the meeting was to gather the child’s opinion, inviting them to step away when the child felt comfortable. However, we noted that in most of the interviews, the parent played a supportive reinforcing role. In some cases, parental support was essential and even helped to focus and clarify ideas. For example, parental assistance proved valuable, particularly in contextualizing items like “making a decision about my care” to the child’s specific situation. This gives an insight into the parent–child dynamic when using a self-report questionnaire like *Advance QoL*. We are confident that *Advance QoL* is relevant, useful, and suitable for young people with advanced cancer. It has been designed to bring the voice of children and adolescents into discussions about their QoL. However, this should also lead us to reconsider how we define a self-report questionnaire aimed for children. Although we developed an instrument specifically intended to collect the perceptions of children without going through a parent or a proxy, we observed that parental support might still be needed in some instances. This observation is possibly due to children’s expectations, the developmental disruptions due to the illness, and parenting habits framed by ongoing cancer treatments [39,40,41]. It is important to take this into consideration, as it also provides insight into the use of self-reported questionnaires in a real-life context outside of a research setting. For some young people, especially the younger ones, the assistance of a parent can enhance their experience with the questionnaire. Parents can enrich and contribute to their children’s reflections. However, despite the possible support, it is still a questionnaire that directly assesses the child’s experience, not that of the parent or proxy.

Finally, the theme “Items requiring thought and response beyond the cognitive capacities of the age group concerned” emerged from the interviewer’s observations. This theme was observed during the interviews with children in response to the questions requiring justification like “Explain why…”. We observed that children’s spontaneous answers were often dichotomous (e.g., “yes/no” or “good/bad”), suggesting the item formulation exceeded the cognitive abilities of the youngest participants. 

The third phase of the project consisted of assessing the social validity of *Advance QoL* as perceived by participants. The results indicate that young people found it acceptable, relevant, useful, clear, and easy to use. They were also satisfied with the questionnaire. In the 8–12 years group, difficulties with language comprehension impaired two aspects of social validity (Supplementary File S1). Misunderstood words or concepts could potentially hinder the tool’s usability, emphasizing the need to address comprehension difficulties. Adolescents generally provided high social validity scores, except for motivation to use the questionnaire. This is explained by concerns about confidentiality and burden raised by some participants. These concerns will need to be anticipated in future implementations of *Advance QoL*. It also underlines both the importance and difficulty of open communication in severe or advanced stages of the illness [42,43]. 

The fourth phase of the project aimed to refine the *Advance QoL* self-reported questionnaires based on the Phase 2 and 3 results. A systematic analysis of the cognitive interviews, combined with social validity data, guided us in determining the best ways to adapt the *Advance QoL* questionnaires. The use of cognitive interviews for questionnaire development led to the emergence of various outcomes for modifying and refining the questionnaires [30]. In our study, most of the comments raised by participants were either recommendations regarding changes in wording or related to the need for additional explanation for certain items. Therefore, the modifications mainly involved removing medical and scientific jargon and instead using words and expressions commonly used by young individuals or adding clarifications to clarify the meaning of items. Thus, all changes made aimed to improve questionnaire understanding and ease of use. Indeed, to optimize adaptation for self-report completion by children and adolescents with advanced cancer, it is essential to minimize barriers and challenges to completion. Finally, the final versions of the questionnaires were approved by the research team members, including young collaborators and a specialized educator from our hospital. 

This study has several strengths. The overall feedback received from Phases 2 and 3 was positive. Clarity, pertinence, and satisfaction were high. The final versions for children (8–12 years) and adolescents (13–18 years) were finally approved by the young collaborators. In our latest interactions, refined versions were enthusiastically received without further suggestions. To our knowledge, this is the first PROM developed to evaluate the QoL of youth with advanced cancer utilizing a bottom-up development strategy to ensure appropriate coverage of life domains identified by final users [21,22]. The *Advance QoL* questionnaires were developed based on a rigorous methodology following suggested guidelines [21,22,23,38,44]. The QoL dimensions had been generated and validated in previous studies conducted specifically with advanced cancer patients [21,22]. Young people with advanced cancer and those undergoing cancer treatments are experts for assessing understanding of *Advance QoL* formulations.

*Advance QoL* aims to collect children’s and adolescents’ own perceptions, which may positively influence clinical discussions and decisions regarding their care and health [12]. Youth involvement in discussions about their interests, choices, and care has positive impacts on their autonomy, self-determination, and empowerment [13,45,46,47], as well as on goal identification and communication [17,18]. In practice, the use of *Advance QoL* would allow clinicians to have access to critical information to guide decisions on care, in line with current pediatric oncology standards of care [8,19,48]. It could also promote better communication between caregivers, patients, and their families [49,50]. 

We should recognize the limitations of the present study. First, while *Advance QoL* is specifically designed for young people with advanced cancer, recruitment challenges led to expanding criteria to include severe conditions from the brain tumour clinic. Cognitive interviews focused on the understanding of the tools and comments were similar across all participants, but we cannot rule out that some of the concerns raised may be influenced by the different contexts of care. Second, to attenuate the burden, we did not use full existing scales to measure social validity but rather selected items from these scales. Although it is theoretically possible that this led to validity issues for the items, all items bore clear face validity on “clarity”, “pertinence”, and “satisfaction”. We also used the CVI to ensure the consistency of responses to the questionnaire. Third, participant awareness of this study’s objective may have influenced the feedback received. The research context might have introduced a response bias. Some may have amplified their appreciation of the tool to please the interviewer. Although particularly pertinent in a young vulnerable population, numerous suggestions for *Advance QoL*’s improvement were provided, contradicting this concern.

## 5. Conclusions

In this study, we elaborated two self-reported versions of *Advance QoL*: QoL instruments for children (8–12 years) and adolescents (13–18 years) with advanced cancer. We collected data through virtual semi-structured cognitive interviews and evaluated the understandability and social validity of the elaborated versions with a group of 12 young patients. The results indicate that the *Advance QoL* self-reported versions were well received and that minimal adjustments were required. The *Advance QoL* tool is now available in three versions (parents/professionals, children 8–12 years old, and adolescents 13–18 years old). Future research should assess its reliability and sensitivity to change, leading to validity and clinical implementation studies. Currently, *Advance QoL* has the potential to offer valuable insights on young people with advanced cancer. 

## Figures and Tables

**Table 1:** Sociodemographic and clinical characteristics of participants.

Participants	Place of Recruitment	Age	Gender	Education Level	Years Since Diagnosis	Type of Cancer
Emma	CHUSJ ^a^	12	Girl	5th grade	3.33	Germinoma
Clara	CHUSJ	12	Girl	6th grade	1.17	Pancreas
Justin	Leucan ^b^	8	Boy	3rd grade	2.75	Medulloblastoma
Marie	CHUSJ	8	Girl	2nd grade	1.25	Medulloblastoma
Alice	CHUSJ	10	Girl	4th grade	2.08	Astrocytoma
Theo	CHUSJ	8	Boy	3rd grade	7.00	Glioblastoma
Beatrice	CHUSJ	14	Girl	Secondary 2	10.00	Acute myeloid leukemia
Emile	Leucan	15	Boy	Secondary 3	1.25	Ewing sarcoma
Alex	CHUSJ	18	Boy	Post-secondary pre-university	6.00	Glioma
Ethan	CHUSJ	18	Boy	Post-secondary pre-university	0.92	Medulloblastoma
Maxime	CHUSJ	13	Girl	Secondary 1	8.00	Neurofibromatosis ^c^
Noa	CHUSJ	17	Boy	Secondary 5	3.75	Neurofibromatosis

^a^ CHU Sainte-Justine. ^b^ A non-profit organization for children with cancer and their families. ^c^ Benign brain tumour treated in the same way as cancer: chemotherapy, radiotherapy and/or targeted therapy.

**Table 2:** Results from the comprehension study of the *Advance QoL* tools (*N* = 12 youths with cancer).

	Themes	Code	Examples of Verbal Statement
Points raised by participant	Problems affecting understanding of the tool	Unclear reference	Beatrice: The question is easy to understand, but what should we write? What’s it in relation to? That’s what I’m wondering. […] The words of the question are easy to understand, but the meaning of the question is more complicated. I don’t really know what to answer [when speaking of the question, “**Has there been a recent situation that is influencing how you feel**?”].
		Misunderstood words and concepts	Clara: It’s correct, but it’s the word “**achievements**” because I understand with the examples, but just the word like that I wouldn’t have understood. Interviewer (I): Can you tell me the few [words] you find most difficult? Theo: Uh, there’s one I can’t remember, it’s too hard to say. […] It’s on the other page. I: Ah, on the other page! Was it “**psychological**”? Theo: Yes
		Instruction too long ^a^	I: Do you want to repeat them in your own words? Justin: Uh what instructions? I: The instructions I just read for the [radar] chart. Justin: Uh we can color inside the shapes. I: Yeah Justin: Well, otherwise I don’t remember. I: Was that a long instruction? Justin: Yes
		Missing item	Beatrice: The question is well understood, but it’s the last day we are speaking of here [when speaking of the question, “**How were your achievements in the last day**?”]. An achievement for me is something a bit bigger, like a wish or a dream, or something you wanted to complete. For me, I see it as something that takes a bit more time or takes several days; it’s not something you do every day like having breakfast. It’s something that requires time or planning. So, on the last day, maybe you haven’t achieved anything today because you’re working to create your achievement, but perhaps in a week, you will have completed your entire achievement. […] I am currently working on my achievements.
		Lack of precision ^b^	Beatrice: […] [When speaking of the question, “**Suggest a way to improve the aspects of your well-being**”]. Would we only answer if we say 0 [poor] or 1 [average], or would we also answer if we say 2 [good]?
	Problems that do not affect understanding of the tool	Sensitive formulation	Emma: “**I adapt well to my illness**”, uh… ((sighs)) I don’t know how to explain it. […] It’s not like we can adapt well to our illness. […] You have to live with it. You’re not well.
		Do not relate to the item ^b^	Noa: There’s one point that I’m less sure about. […] The point would be “**to have good relationships with nurses, doctors, and caregivers**”. […] Well, generally, I think that fits in, but if we take an example of someone who might find it a bit more difficult to talk to adults or older people, there could be a way to remove that point and, in my opinion, have a pretty good social well-being. […] It could be important, but at the same time, we could still take it out and it would still work.
		Too many examples ^a^	Clara: I find that for me there are too many examples. […] Just two, three would be better to understand. […] I wouldn’t have added other things, I would have removed things because it’s too much. […] Because for an example, you don’t really need a lot of things. Then, in most of my notebooks, an example is just a sentence, so it’s not very long.
	Modifications to improve and enhance the tool	Format-related suggestions	Emile: In the middle of the [radar] chart, it says “well-being”? […] Why is it there? […] It makes me think that if you put all 0, “all poor”, it circles well-being. […] Maybe instead you could just put wellbeing in the title where it says, “Your turn”. You write wellbeing and then frame it with the whole image.
		Content-related suggestions	Emile: There are a lot of people who don’t want to give their information to everyone for free. They’re not going to say the big points of why they’re sad and all that. What you could do is mention either when you give the sheet or write on it that the information is just going to be read by the doctors or something like that. […] Even, even to the parents, there are children who don’t want to say things to their parents, who keep things private from their parents, then who don’t want to tell them, they are not yet ready to admit it to their parents. […] Otherwise, I have an idea, put on the sheet […] a little case uhm “tick in boxes” like just show the doctors, show the nurses, or show the parents. So, if the child just wants to show to the doctors, check doctor, if they want doctors, nurses and uh parents, they check all. We want to know who’s reading our life, our private life. Emma: I’d like to add something. […] To have fun in the playroom [at the hospital] […]. I: Okay, when you could do that in your day, for example, did you have fun? Emma: Yes, yeah. […] Let’s say it’s my only pleasure when I’m in the hospital.
	Positive features of the tool	Examples	Emile: What I like is that you give ideas. It’s not just physical well-being you give like pain, energy, slept well, breathing well then nausea, vomiting. “Explain your answer”, you can use that. You can say “oh yes it was good”, but you can say like “oh yes it was good because I slept well, and I breathe well”.
		Time window ^a^	I: What do you think about “**the last day**”? Alice: It’s good because it’s not too far, you kind of know what happened. You remember it well, then it’s easy to answer the questionnaire when you remember your day.
		Organization of instructions	Alice: I think it’s good because, if you’ve forgotten something, you can always reread [the instruction on page 2] to get ideas back in your head about what you need to do. I: Ok then, do you find it useful? Alice: Yeah Beatrice: I think it’s good, I like the fact that we have like steps, we have one and two with what we need to do.
		Measurement scale	Noa: I find that it makes it a little more helpful because I’m taking the example of certain people who aren’t necessarily too uhm, who don’t find it too easy to express what they feel, so I find that it helps that he has 3 choices
Elements observed by the interviewer	Item requiring thought and response beyond the cognitive capacities of the age group concerned ^a^		I: Would you know what to answer here? Justin: No Parent: You said your physical well-being was fine? Why did you say that? Justin: Because it wasn’t bad

^a^ Results from children’s interviews (8–12 years old) only. ^b^ Results from teenager’s interviews (13–18 years old) only.

**Table 3:** Results of the social validity study.

Item ^a^	Children (*N* = 6)			Adolescents (*N* = 6)			Total Sample (*N* = 12)		
	Median	Min	Max	Median	Min	Max	Median	Min	Max
I understand what is being asked.	4.75	4	5	5	4	5	5	4	5
2. The tool is easy to use.	5	3	5	5	4.5	5	5	3	5
3. The tool is useful for assessing my well-being.	4.25	4	5	5	4	5	5	4	5
4. The length of the questionnaire is acceptable.	5	4	5	5	5	5	5	4	5
5. The tool is appropriate for assessing the well-being of other young people with cancer like me.	5	4	5	5	5	5	5	4	5
6. The tool allows me to describe what’s not going so well in my life.	5	4	5	5	4	5	5	4	5
7. There are disadvantages to completing the tool ^b^.	4	3	5	5	4	5	5	3	5
8. I liked the tool.	4.75	4	5	5	5	5	5	4	5
9. I recommend using the tool.	4.25	4	5	5	5	5	5	4	5
10. I am motivated to use the tool.	5	4	5	4	3	5	5	3	5
Total	4.88	3	5	5	3	5	5	3	5

^a^ Measurement scale ranges from 1 (Totally disagree) to 5 (Totally agree). ^b^ Reversed-coded item.

**Table 4:** Examples of changes made to *Advance QoL* based on feedback received (Phases 2 and 3).

	Feedback Received	Initial Wording	Changes Made
*Advance QoL*—8–12 years version	Unclear reference	Here, you can write down an event you’d like to share.	Item originally from the parent/healthcare professional version to contextualize the assessment: “Situation/clinical context that may affect the evaluation of the QoL”. Item **removed** as it was judged unclear and redundant with the rest of the questionnaire.
	Misunderstood words and concepts	My other symptoms are relieved. (Nausea, vomiting, diarrhea, etc.)	The word “symptom” is derived from medical jargon, difficult for some young people to understand. The word “symptoms” was removed from the questionnaire and replaced by easy, concrete examples. **“I do not have a stomachache or a headache.”**
	Sensitive formulation	I adapt well to illness.	“Adapt well” was felt inadequate in the context of advanced cancer. We changed this into a phrase more respectful of the diverse paths and journeys of individuals. **“I feel well-adapted.”**
*Advance QoL*—13–18 years version	Missing item	Achievements are when: - I do a project or activity that I wanted to do. - I fulfill a wish or dream.	According to the feedback received, two elements were missing: (1) the notion of “working to achieve something” or “being in the process of doing a project” and (2) achieving something that makes you feel “fulfilled, satisfied and happy”. **“Achievements:** - **I do activities that I wanted to do.** - **I am doing something that makes me happy.** - **I am doing a project.** - **I fulfill a wish or dream.”**
	Lack of precision and sensitive formulation	Suggest a way to improve the aspects of your well-being.	Following feedback, we added the idea of maintaining these aspects of well-being so that young people who already mentioned having a good well-being to the items feel considered. The formulation was adapted to generate ideas and remove the mandatory tone of the initial phrase. **“Ideas for improving or maintaining these aspects of your well-being.”**

## Data Availability

The data presented in this study are available on request from the corresponding author.

## Supplementary Materials

The following supporting information can be downloaded at https://www.mdpi.com/article/10.3390/curroncol31040170/s1, File S1: Interview guide; Table S1: Result of the content validity index (CVI). Reference [36] is cited in the Supplementary Materials.
