# Supplementary material for: Quality-of-Life Assessment in Pediatric Advanced Cancer: Development of the Patient-Reported Outcome Measure Advance QoL
Source: Curr Oncol. 2024 Apr 19;31(4):2289–304. doi: 10.3390/curroncol31040170 (PMC11049209; doi:10.3390/curroncol31040170)
Supplement: Supplementary file 1 [file curroncol-31-00170-s001.zip › curroncol-2953672-supplementary.pdf]

Supplementary material A

File S1. Interview guide

Semi-structured cognitive interview

*Note: Verbal and para-verbal language (Mmm, okay, I see, etc.) will be used to encourage the participant to express his or her point of view.

<u>For each questionnaire item</u>

- What does that mean to you? / What did you understand from the question? / Could you tell me in your own words what is required?

- Are there any words or expressions that are difficult to understand? / Are there any expressions that are unclear? / Are there any words or expressions you think we should avoid?
    - What don't you understand? / What makes it difficult? / What could be replaced to make it easier to understand?
    - What would help you understand better?

- Do the examples help you better understand what is being asked?

<u>Measurement scale</u>

- Version for 8-12 years: Is the use of the smiley faces clear to you?
- Version for 13-18 years: Is the "bad, average and good" scale clear to you?

<u>Recall period</u>

- What do you think of using the 24-hour period (in the last day) to fill in the tool?
- Do you have any suggestions for improvement?

Supplementary material B

Table S1. *Result of the content-validity index (CVI)* [a].

	Children (N = 6)			Adolescents (N = 6)		
Item	Agreement	I-CVI	UA [b]	Agreement	I-CVI	UA
1. I understand what is being asked.	6	1	1	6	1	1
2. The tool is easy to use.	4	0.67	0	6	1	1
3. The tool is useful for assessing my well-being.	6	1	1	6	1	1
4. The length of the questionnaire is acceptable.	6	1	1	6	1	1
5. The tool is appropriate for assessing the well-being of other young people with cancer like me.	6	1	1	6	1	1
6. The tool allows me to describe what's not going so well in my life.	6	1	1	6	1	1
7. There are disadvantages to completing the tool [c].	4	0.67	0	6	1	1
8. I liked the tool.	6	1	1	6	1	1
9. I recommend using the tool.	6	1	1	6	1	1
10. I am motivated to use the tool.	6	1	1	5	0.83	0
**S-CVI/Ave**		0.93			0.98	
**S-CVI/UA**			0.8			0.9
**Average proportion of items judged as relevance across the experts**	0.93			0.98		

[a] Based on Yusoff (2019). *Education in Medicine Journal* 11(2): 49-54.

[b] Universal agreement.

[c] Reversed-coded item.
